# Circulating microRNAs and endometriosis: a comprehensive analysis and validation of identified biomarkers in an Indian population

**DOI:** 10.1530/RAF-25-0019

**Published:** 2025-10-16

**Authors:** Shivangi Chauhan, Ashutosh Halder, Mona Sharma, Jai Bhagwan Sharma, Deepak Pandey, Neeraj Kumar

**Affiliations:** ^1^ Department of Reproductive Biology, All India Institute of Medical Sciences, New Delhi, India; ^2^ Department of Obstetrics and Gynecology, All India Institute of Medical Sciences, New Delhi, India

**Keywords:** endometriosis, miRNA, biomarkers, plasma, qRT-PCR

## Abstract

**Graphical abstract:**

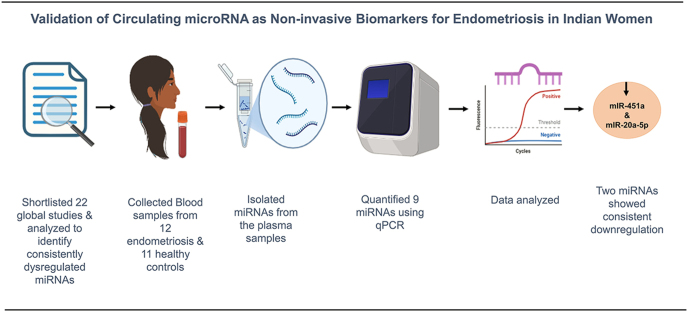

**Abstract:**

Endometriosis is a prevalent condition where tissue similar to the uterine lining grows outside the uterus, causing pain and infertility. Diagnosing endometriosis typically requires invasive procedures such as laparoscopy. MicroRNAs (miRNAs) have emerged as promising noninvasive biomarkers for various diseases, including endometriosis. However, studies have shown inconsistent miRNA expression patterns across populations. This study aims to validate circulating miRNAs as biomarkers for endometriosis in Indian women, addressing the limited validation data available for this population. This comprehensive review identified nine circulating miRNAs based on reproducibility and consistent expression patterns. Women with advanced-stage endometriosis (*n* = 12) and controls (*n* = 11) were recruited. Plasma samples were collected based on clinical symptoms, CA-125 levels, ultrasound, MRI findings, and laparoscopic confirmation. miRNA expression was quantified using qRT-PCR, and receiver operating characteristic (ROC) analysis was performed to assess diagnostic potential. Nine miRNAs (miR-451a, let-7b, miR-150-5p, miR-17-5p, miR-3613-5p, miR-20a-5p, miR-342-3p, miR-125b-5p, and miR-21-5p) were analyzed. Among them, miR-451a and miR-20a-5p exhibited significantly lower expression in endometriosis patients (*n* = 12) compared to controls (*n* = 11). ROC analysis demonstrated promising diagnostic potential for these miRNAs. miR-451a showed distinct trends compared to previous studies, while miR-20a-5p was consistent with earlier research. Although encouraging, these findings are based on a limited sample size. Larger multicenter studies across diverse populations using reliable reference genes are needed to fully assess the diagnostic value of these miRNAs as biomarkers for endometriosis.

**Lay summary:**

Endometriosis is a common condition where tissue similar to the uterine lining grows outside the uterus, causing pain and infertility. Diagnosing it usually requires invasive procedures such as laparoscopy. We focused on microRNAs (miRNAs), small molecules in plasma that could offer a noninvasive way to diagnose endometriosis. After reviewing 45 research articles, we identified 102 miRNAs that were elevated and 197 that were reduced in endometriosis patients. From these, we selected nine promising miRNAs for validation in the Indian population. We collected blood samples from 12 women with endometriosis and 11 healthy controls. Our analysis showed significant differences in miRNA expression, with miR-451a and miR-20a-5p showing strong potential to distinguish between endometriosis patients and healthy individuals. These findings suggest that miRNAs could improve the diagnosis of endometriosis in a less invasive manner. In conclusion, our research highlights the potential of miRNAs in advancing endometriosis diagnosis and management.

## Introduction

Endometriosis is a prevalent gynecologic disorder that affects approximately 10% of reproductive-aged women globally; however, in India, its prevalence is notably high, estimated at 25–35% in women of reproductive age (Ragab *et al.* 2015, Umelo 2017, Moradi *et al.* 2021). It is a hormone-driven disease primarily characterized by pelvic pain, dysmenorrhea, and infertility resulting from the abnormal growth of endometrial tissues outside the uterus (Verkauf 1987, Giudice & Kao, 2004, Nezhat *et al.* 2024). Early diagnosis is often delayed by 5–10 years because symptoms are nonspecific (Greene *et al.* 2009, Hudelist *et al.* 2012). Recently, the Endometriosis Risk Advisor (EndoRA) mobile application, using an AI-based algorithm, has been introduced (Nezhat *et al.* 2023). Although it shows high sensitivity, its specificity is limited and it cannot replace a definitive diagnosis. Conventional imaging, such as ultrasound and magnetic resonance imaging (MRI), is inadequate for detecting and staging the disease. At present, laparoscopy remains the gold standard, but it is expensive, invasive, and carries surgical risks (Simoens *et al.* 2007, Soliman *et al.* 2017). Early detection is crucial for effective intervention, necessitating a noninvasive test for specific biomarkers. Despite multiple efforts to identify suitable biomarkers, a definitive choice remains elusive because they do not demonstrate adequate sensitivity or specificity (May *et al.* 2010, 2011, Fassbender *et al.* 2014).

In recent years, various omics-based approaches have been employed to advance the understanding and diagnosis of endometriosis. Genomics has provided insights into genetic predisposition and regulatory elements associated with the disease pathophysiology (Taylor *et al.* 2002). Proteomics has been instrumental in identifying protein-level alterations in eutopic and ectopic endometrial tissues (Azeze *et al.* 2024), while metabolomics has revealed distinct metabolic signatures in patients with endometriosis, reflecting underlying inflammatory and hormonal imbalances (Pandey 2024). Despite these advances, each of these approaches faces challenges such as sample heterogeneity, dynamic expression profiles, and complex biofluid matrices. In contrast, circulating microRNAs (miRNAs) offer several advantages: they are highly stable in body fluids, exhibit disease- and tissue-specific expression patterns, and can be noninvasively detected, making them promising minimally invasive biomarkers for both diagnosis and monitoring of endometriosis. MicroRNAs (miRNAs) have gained interest as possible biomarkers for various diseases, including endometriosis, because of their occurrence in diverse extracellular biological fluids such as plasma (Arroyo *et al.* 2011), serum (Chen *et al.* 2008), saliva (Gallo *et al.* 2012, Bendifallah *et al.* 2022), and urine (Mall *et al.* 2013), among others (Weber *et al.* 2010). miRNAs are short, 19–24 nucleotide-long noncoding RNAs that can be released into circulation and safeguarded from endogenous RNase degradation due to inclusion in exosomes or binding with specific protein complexes (Bayraktar *et al.* 2017, Thomou *et al.* 2017). With over 2,600 miRNAs present in the human genome, each exhibiting distinct expression profiles linked to diverse diseases (Min & Chan, 2015, Chakraborty & Das 2016, Srivastava *et al.* 2017), investigations have explored irregular miRNA expression within endometrial lesions (Wei *et al.* 2015). Moreover, these circulating miRNAs in serum (Wang *et al.* 2013, 2016, Cosar *et al.* 2016) and plasma (Suryawanshi *et al.* 2013, Vanhie *et al.* 2019) have been studied extensively, as alterations in their concentrations within the bloodstream could signify shifts during regular physiological processes (Sredni *et al.* 2011, Lai *et al.* 2014) and have been correlated with various pathological states, including gynecologic disorders.

Despite substantial research aimed at constructing a blood-based miRNA signature for detecting endometriosis patients, no novel blood-based biomarkers have been integrated into clinical practice for diagnosing endometriosis (Nisenblat *et al.* 2016, Leonova *et al.* 2021). Although certain serum markers have been identified, their applicability is hindered by challenges related to sensitivity, specificity, and reproducibility. These hurdles may stem from differences in study design, analytical methods, sample processing, storage, quantification/sequencing techniques, variability in endometriosis phenotypes, ethnicity, selection of reference genes for data normalization, and statistical approaches (Agrawal *et al.* 2018, Williams *et al.* 2019, Monnaka *et al.* 2021).

Despite extensive research on endometriosis-associated miRNAs, only limited reports exist that comprehensively compare the findings across multiple laboratories and subsequently validate them. Therefore, the objective of this study was to thoroughly assess the identified circulating endometriosis-specific miRNAs present in peripheral blood and to validate the most consistently reported candidates in the context of India, where such investigations are limited.

## Materials and methods

### Data search and analysis

A comprehensive literature search encompassing publications up to December 16th, 2022, was conducted across electronic databases and the scientific literature platforms, including PubMed and ArrayExpress. The search utilized keywords such as ‘biomarker’, ‘endometriosis’, ‘microRNA’, ‘noninvasive diagnosis’, ‘miRNA’, and ‘qRT-PCR’, both individually and in various combinations. For each study included in the analysis, essential details were accurately extracted and concisely summarized. These details encompassed microRNA expression variances, sample origins, sample sizes, quantification methods employed, and the validation techniques utilized.

Two comprehensive compilations were generated: one included all identified microRNAs from the included studies, while the other comprised only the microRNAs subjected to validation. These inventories offer a comprehensive perspective on the microRNAs examined within the domain of endometriosis. Composite scores were calculated based on the frequency of each miRNA’s mention across multiple studies. For each study, a plus sign (+) indicates significantly upregulated miRNAs, a minus sign (−) denotes downregulated miRNAs, and a hash sign (#) represents miRNAs with no significant change. The number preceding each sign indicates the number of studies reporting that observation (Supplementary File S2 (see section on Supplementary materials given at the end of the article)). Separate cumulative scores were calculated for upregulated, downregulated, and unchanged miRNAs within both the screening and validation study categories, based on the number of studies in which each miRNA was consistently reported. miRNAs consistently reported with the same direction of expression (either up or down) were prioritized. To assess directional consistency, we identified overlaps among replicated differentially expressed miRNAs and flagged those with contradictory expression patterns across studies. miRNAs showing directional conflicts but with high absolute scores (e.g., multiple +1s, −1s, or #1s) were further examined to compare trends between screening and validation datasets. Greater weight was assigned to miRNAs with a consistent expression direction across both study types. In cases of conflicting results, particular emphasis was placed on validated miRNAs demonstrating stable expression trends across diverse populations.

A higher score denoted heightened representation of a specific microRNA across studies. Employing these composite scores, a conclusive compilation of the foremost differentially regulated microRNAs was precisely curated. This method facilitated a comprehensive investigation directed toward identifying promising microRNAs as potential markers for endometriosis. The integration of validated miRNAs displaying consistent expression patterns across multiple studies aimed to enhance the reproducibility and applicability of the findings.

### Study population and sample collection

**Figure 1:**
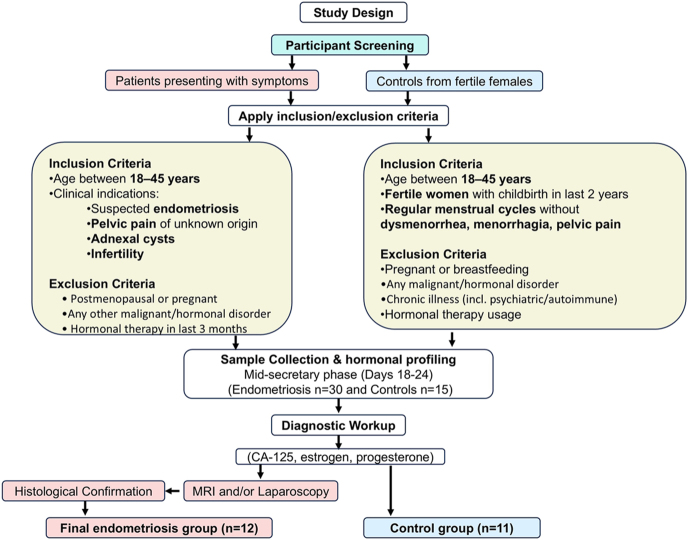
Flowchart of participant recruitment and selection. This flowchart summarizes the stepwise screening of 30 patients with suspected endometriosis and 15 healthy controls. After applying clinical, imaging, and histological criteria, 12 patients with confirmed endometriosis and 11 eligible controls were included in the final analysis. Inclusion and exclusion criteria were strictly applied to ensure clinical and demographic relevance in both groups.

Initially, participant selection was based on a comprehensive range of criteria, including indications such as suspected endometriosis, pelvic pain with undetermined origin, the presence of adnexal cysts, or infertility issues. Notably, individuals in the postmenopausal phase and pregnant subjects were excluded. Furthermore, participants with a medical history encompassing cancer of any type, hysteromyoma, adenomyosis, endometrial synechiae, polycystic ovary syndrome, hydrosalpinx, or any hormonal or malignant disorders were ineligible for the study. To prevent potential therapy-related effects on miRNA secretion, participants who used any form of hormonal therapy in the last 3 months were excluded from this study. The overall recruitment, screening, and final selection of participants for both patient and control groups are outlined in the study flowchart (Fig. 1).

This schematic representation illustrates the stepwise application of inclusion and exclusion criteria, initial enrollment, diagnostic confirmation by imaging and laparoscopy, and the eventual classification into study and control cohorts. Based on the inclusion and exclusion criteria (Fig. 1), an initial cohort of 30 patients and 15 healthy controls was recruited. Blood samples were collected from all participants during the mid-secretory phase of the menstrual cycle (days 18–24). Plasma samples were assessed for CA-125, estradiol, and progesterone to evaluate the hormonal milieu for all participants. Following clinical evaluation and diagnostic workup – including MRI (performed in five patients before laparoscopy) and laparoscopic visualization with histological confirmation – 12 patients with confirmed endometriosis were included in the final analysis. This group also comprised three individuals with recurrent disease, each of whom had undergone laparoscopic surgery at least 4 years earlier. In addition, eleven age-matched controls were identified, based on specific inclusion criteria: fertile females who had given birth within the last 2 years and who were not pregnant at the time of sample collection. Laparoscopy was considered inappropriate for ethical reasons, and participants needing such procedures for gynecological conditions were excluded to ensure healthy controls. The exclusion criteria for controls were that they lacked any gynecological disorders, were not pregnant or breastfeeding, and had no history of cancer, hysteromyoma, adenomyosis, endometrial synechiae, PCOS, hormonal diseases, or substance abuse. In addition, individuals with chronic medical conditions, psychiatric disorders, or autoimmune disorders, who were receiving hormonal therapy, or who were unwilling to provide consent, were excluded. The control group included participants aged 22–37 years, while the study group included participants aged 24–39 years, and the study adhered to a stringent selection process to ensure the clinical and demographic relevance of the participants.

### Plasma collection and quality assessment

Five milliliters of whole blood was collected from each participant in EDTA tubes before the surgical intervention. The collected blood specimens were promptly transported to the laboratory within thirty minutes for subsequent processing. The plasma was isolated from the whole blood after centrifugation at 1,900 ***g*** for ten minutes at 4°C. Hemolysis in plasma samples was visually identified by observing the color of the supernatant plasma. Furthermore, a quantitative assessment was conducted by measuring the absorbance at 415 nm using a spectrophotometer (Thermo Scientific Nanodrop), and samples with values less than 0.2% were selected for RNA extraction. The separated plasma was then divided into aliquots, each containing 200 microliters, and preserved at a temperature of −80°C for storage.

### RNA extraction and reverse transcription

Two hundred microliters of plasma was subjected to total RNA extraction using the QIAzol Lysis Reagent, following the guidelines provided by the manufacturer (Qiagen, USA). The RNA purity was evaluated by measuring the A260/A280 ratio with a Nanodrop 2000. Moreover, a spiked-in synthetic template miRNA (cel-miR-39) from Qiagen was introduced alongside QIAzol. This addition served to evaluate the efficacy of the RNA isolation, cDNA synthesis, and PCR amplification processes. High-quality RNA samples were used to synthesize complementary DNA (cDNA) using the miRCURY® LNA® miRNA SYBR® Green PCR Kit (Qiagen). The relative expression of miRNAs was determined by SYBR Green Quantitative Real-Time Polymerase Chain Reaction (qRT-PCR) using the miRCURY® LNA® RT Kit (Qiagen). Cel-miR-39 and UniSP6 were used for the extraction and template control processes, respectively. miR-16-5p was used as an endogenous control to determine relative miRNA expression levels. The relative mRNA level was calculated using the formula 2^(−ΔCT).

### Statistical analysis

The data were analyzed using MS Excel and GraphPad Prism v. 8.01. Student’s *t*-test was used to compare clinical characteristics between the endometriosis and control groups, as the data were normally distributed and are expressed as the mean ± standard deviation (SD). The Mann–Whitney U test was used to compare miRNA expression levels, and the results are presented as medians and interquartile ranges (IQR). Post hoc power analysis was performed using the online calculator available at ClinCalc (https://clincalc.com/stats/power.aspx, accessed on July 4, 2025), based on observed effect sizes, sample sizes, and a significance level of 0.05. ROC analysis was performed to assess the diagnostic value of each miRNA biomarker, and the area under the ROC curve (AUC) was calculated. A *P*-value of <0.05 was considered to indicate statistical significance. Furthermore, to account for multiple testing, the false discovery rate (FDR) was controlled using the Benjamini–Hochberg procedure (*q* = 0.05). Adjusted *P*-values were calculated with the online tool available at https://tools.carbocation.com/FDR. Binary logistic regression was performed using SPSS (Norusis 2008) to assess the predictive value of miR-20a-5p and miR-451a for distinguishing endometriosis cases from controls. The dependent variable was binary (group: 0 = control, 1 = endometriosis), and both miRNAs were entered simultaneously as continuous independent variables using the Enter method. Predicted probabilities were saved and used to generate the ROC curve. Model significance was evaluated using the Omnibus Test of Model Coefficients (*χ*^2^ test), and classification performance was assessed by sensitivity, specificity, and area under the ROC curve (AUC).

## Results

### Data search

A total of 45 articles discussing circulating miRNAs in blood (plasma/serum) were identified through electronic databases and the scientific literature searches. After a thorough assessment, 23 articles were excluded. These exclusions were due to duplicate titles, secondary research articles, and miRNA studies related to other malignancies in addition to endometriosis. A total of 22 studies that met our inclusion criteria were identified. These investigations, primarily originating from China (*n* = 6), the USA (*n* = 5), Iran (*n* = 3), and other European and Asian countries, were conducted with an almost equal distribution of plasma and serum samples (Table S1). However, it is noteworthy that India, despite having a substantial population and a high prevalence of endometriosis, was not represented in the selected studies.

### Differential expression of circulating miRNAs

**Figure 2:**
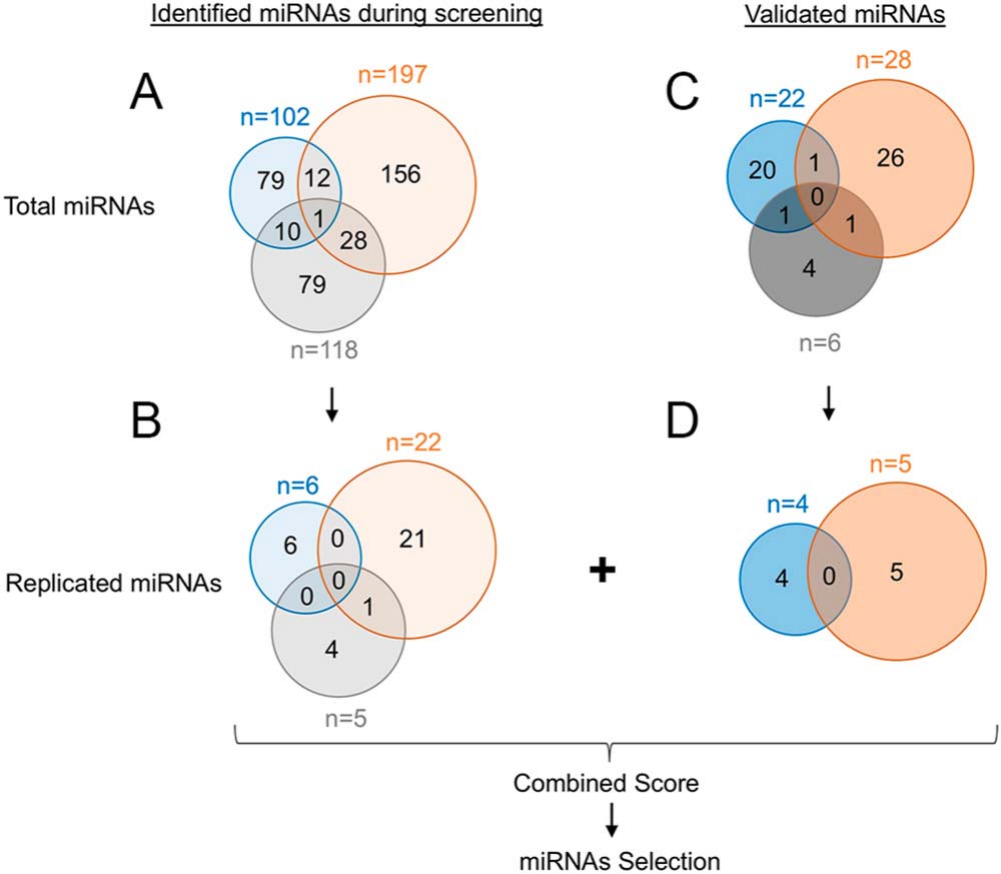
Analysis of miRNAs in blood plasma or serum samples of endometriosis patients. (A) Total identified miRNAs, (B) identified miRNAs replicated in two or more studies, (C) total validated miRNAs, and (D) validated miRNAs replicated in at least two studies. Blue represents upregulated miRNAs, orange represents downregulated miRNAs, and gray represents unchanged or undefined miRNAs. A plus sign (+) indicates significantly upregulated miRNAs, a minus sign (−) denotes downregulated miRNAs, and a hash sign (#) represents miRNAs with no significant change. The number preceding each sign indicates the number of studies reporting that observation.

In the analysis of 22 studies, 102 upregulated miRNAs, 197 downregulated miRNAs, and 118 miRNAs with unaltered or undefined status (NA) were identified (Table S2). Remarkably, only 13 miRNAs were common to both the upregulated and downregulated groups (Fig. 2A). This suggests that most of these miRNAs either consistently trend across multiple studies or are unique to specific studies. The application of the reproducibility criterion, which necessitated confirmation in at least two studies, revealed that most of the identified miRNAs lacked this confirmation and were identified in single studies. Consequently, confirmation was achieved for only 6 upregulated miRNAs, 22 downregulated miRNAs, and 5 miRNAs with unaltered or undefined status (Fig. 2B). This implies that only 28 out of the 286 (10%) significantly differentially expressed miRNAs were detected in at least two studies (Fig. 2A and B). Similarly, in the analysis of miRNAs validated in various laboratories worldwide, only four upregulated and five downregulated miRNAs were identified as being replicated by more than one laboratory, accounting for 18% (9/49) of the miRNAs in the replication category (Table S3, Fig. 2C and D). A combined list of both identified and validated miRNAs has been prepared that replicated in at least two studies (Table S4). Subsequently, we evaluated the expression direction in both the identified and validated (replicated) miRNA groups. A combined score was then computed, with preference given to validated miRNAs in cases where two miRNAs received the same score. This process resulted in the selection of nine miRNAs from the initial pool, making them promising candidates for future investigations in endometriosis (Table 1).

**Table 1:** List of selected miRNAs.

Name	Scoring (total)		Accession no.
	Identified	Validated	
Selected miRNAs			
miR-451a	3↑	2↑	MIMAT0001631
Let-7b-5p	3↓	2↓	MI0000063
miR-150-5p	2↑, 1↔	2↑	MIMAT0000451
miR-17-5p	2↓, 2↔	2↓	MIMAT0000070
miR-3613-5p	2↓, 1↔	2↓	MIMAT0017990
miR-20a-5p	1↓, 2↔	2↓	MIMAT0000075
miR-342-3p	2↑, 1↓	2↑	MIMAT0000753
miR-125b-5p	1↑, 2↓	2↑, 1↔	MIMAT0000423
miR-21-5p	2↓	1↓, 1↔	MIMAT0000076
Endogenous reference			
miR-16	-	-	MIMAT0000069

↑Indicates significantly upregulated miRNAs; ↓denotes downregulated miRNAs; ↔ represents miRNAs with no significant change. The number preceding each sign indicates the number of studies reporting that observation.

### Study subjects

Based on the participant selection criteria outlined above, a total of 23 individuals were included in the final analysis: 12 participants were diagnosed with endometriosis through laparoscopic evaluation, while 11 were classified as healthy controls. Comprehensive diagnostic, clinical, and relevant subject characteristics are detailed in the supplemental file (Table S5).

No statistically significant difference in age between the study groups was observed (*P* = 0.1663). Among the women in the endometriosis group, seven reported experiencing chronic pelvic pain, three exhibited symptoms of both pelvic pain and infertility, and two described experiencing painful and burning micturition. Furthermore, among the patients who presented with clinical symptoms, nine had been experiencing them for a period ranging from 3 to 7 years, while three had been symptomatic for 2 years. Two patients with a 7-year history of recurrent endometriosis were included. Samples were collected during the presumed luteal phase based on patient-reported menstrual history; however, precise phase determination was limited in some participants due to irregular menstrual cycles. Plasma progesterone and estrogen levels were assessed to approximate luteal phase status. Conversely, all the healthy control subjects included in the study displayed regular menstrual cycles devoid of any discernible gynecological pathology. The mean serum CA-125 level was markedly elevated in the endometriosis group (51.9 ± 9.5 U/mL) compared to controls (16.0 ± 8.0 U/mL), showing a highly significant difference (*P* < 0.0001). However, serum estradiol (E2) and progesterone levels measured during the presumed mid-secretory phase were comparable between groups. Estradiol averaged 139.7 ± 36.9 pg/mL in the endometriosis group versus 144.1 ± 34.1 pg/mL in controls (*P* = 0.7689), while progesterone levels were 10.3 ± 2.9 and 10.6 ± 3.2 ng/mL, respectively (*P* = 0.8557) (Table S5). These findings suggest that while CA-125 is a reliable serum marker to distinguish endometriosis from asymptomatic individuals, circulating levels of estradiol and progesterone may not differ significantly in the mid-luteal phase between groups. In addition, none of the healthy controls had undergone any surgical procedures other than cesarean delivery (4 out of 11 control participants), and these deliveries had occurred at least 2–3 years before sample collection.

### Differential expression analysis

**Figure 3:**
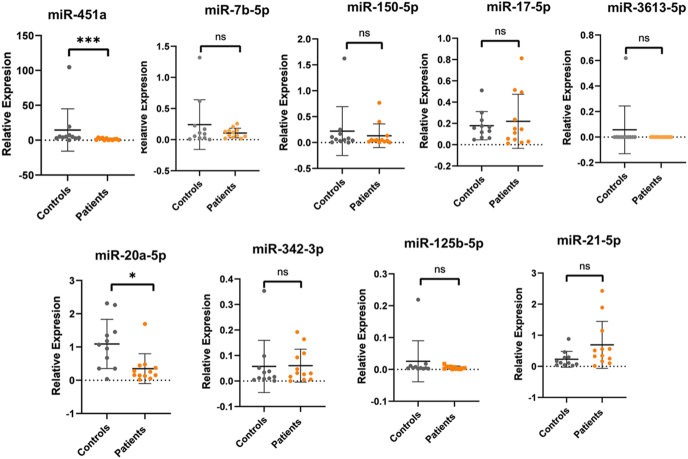
Expression levels of nine miRNAs, normalized to those of miR-16-5p. The median and interquartile range (IQR) are displayed on a dot plot. According to the Tukey method, whiskers and outliers are presented at locations where the IQR is 1.5 times or less (25th percentile minus IQR or 75th percentile plus IQR), with points outside of this range plotted individually. Statistical significance is indicated by asterisks based on *P*-values: ns (not significant) = *P* > 0.05, * = *P* ≤ 0.05; ** = *P* ≤ 0.01, *** = *P* ≤ 0.001.

In this case-control study, patients with well-characterized advanced-stage endometriosis and matched controls were included. The relative expression levels of nine miRNAs (miR-451a, let-7b, miR-150-5p, miR-17-5p, miR-3613-5p, miR-20a-5p, miR-342-3p, miR-125b-5p, and miR-21-5p) were quantified in plasma samples via quantitative real-time polymerase chain reaction (qRT-PCR). Patients with endometriosis showed lower expression levels of miR-451a and miR-20a-5p than the control group. Post hoc power was estimated at 99.4% for miR-451a (after one outlier adjustment in the control group) and 82.2% for miR-20a-5p. No statistically significant differences in expression were detected for the remaining miRNAs (Fig. 3).

### ROC curve analysis

**Figure 4:**
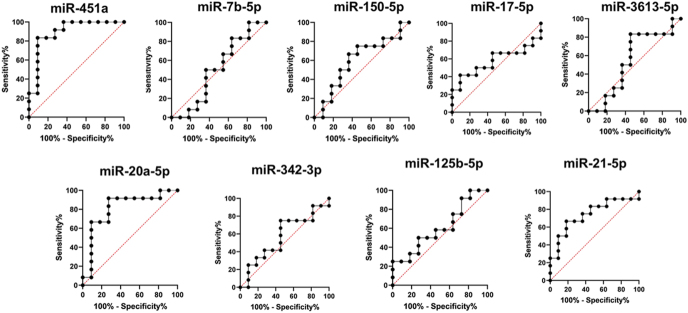
Receiver operating characteristic (ROC) curve analysis of plasma miR-451a, let-7b, miR-150-5p, miR-17-5p, miR-3613-5p, miR-20a-5p, miR-342-3p, miR-125b-5p, and miR-21-5p. The areas under the ROC curves for miR-451a and miR-20a-5 were 0.8939 and 0.8106, respectively.

Receiver operating characteristic (ROC) curve analysis was performed to evaluate the discriminatory power of plasma miRNAs that were differentially expressed between endometriosis patients and controls. Our study revealed that miR-451a and miR-20a-5p exhibited a promising diagnostic performance, with an area under the curve (AUC) value of 0.8939 (95% confidence interval (CI) = 0.7508 to 1.000) and 0.8106 (95% confidence interval (CI) = 0.6164–1.000), respectively (Fig. 4). The sensitivity and specificity values of miR-451a (83.33 and 90.91% at a cutoff <2.790) and miR-20a-5p (91.67 and 72.73% at a cutoff <0.5766) collectively indicate their potential as diagnostic biomarkers for endometriosis in the Indian population (Table 2). Among the nine candidate miRNAs tested, miR-451a (*P* = 0.0007) and miR-20a-5p (*P* = 0.0106) showed significantly lower expression in endometriosis patients compared to controls. After correction for multiple testing using the Benjamini–Hochberg procedure (*q* = 0.05), both miR-451a (*P*_adj = 0.0063) and miR-20a-5p (*P*_adj = 0.0477) remained statistically significant, confirming their potential as circulating biomarkers (Table 2).

**Table 2:** ROC analysis of individual miRNAs. Values in bold represent statistically significant results.

miRNA	Area	95% CI	*P* value			Cutoff	Sensitivity	Specificity
			ROC	MWT	BH adj			
miR-451a	**0.8939**	0.7508–1.000	0.0014	**0.0007**	**0.0063**	<2.790	83.33	90.91
let-7b-5p	0.5076	0.2559–0.7592	0.9509	0.9759	0.9759	>0.1011	50.00	54.55
miR-150-5p	0.5985	0.3571–0.8398	0.4237	0.4491	0.7810	<0.05153	66.67	63.64
miR-17-5p	0.5758	0.3300–0.8215	0.5383	0.5658	0.7810	<0.1539	66.67	54.55
miR-3613-5p	0.553	0.2993–0.8067	0.6666	0.6947	0.7815	<0.0003183	83.33	54.55
miR-20a-5p	**0.8106**	0.6164–1.000	0.0116	**0.0106**	**0.0477**	<0.5766	91.67	72.73
miR-342-3p	0.5682	0.3252–0.8112	0.5796	0.6075	0.7810	>0.01695	75.00	54.55
miR-125b-5p	0.6061	0.3707–0.8414	0.3889	0.4042	0.7810	<0.004757	58.33	54.55
miR-21-5p	0.7424	0.5330–0.9519	0.0489	0.0512	0.1536	>0.313	66.67	81.82

MWT, Mann–Whitney test; BH adj, Benjamini-Hochberg adjusted; CI, confidence interval.

Furthermore, a binary logistic regression was conducted to evaluate whether circulating levels of miR-20a-5p and miR-451a were predictive of endometriosis status. The model was statistically significant, *χ*^2^(2) = 20.54, *P* < 0.001, indicating that the two miRNAs collectively distinguished between patients and controls. The model explained 78.8% of the variance in disease status (Nagelkerke *R*^2^ = 0.788) and correctly classified 87.0% of cases (81.8% controls, 91.7% patients) (Fig. S2). The Hosmer–Lemeshow test was non-significant (*P* = 0.995), suggesting good model fit.

## Discussion

To the best of our knowledge, this study represents the first of its kind conducted in India, where we compared all available circulating miRNA data and verified the most consistent miRNA expression in this population. Here, we evaluated the expression levels of nine selected miRNAs as potential biomarkers for endometriosis. Significant variations in the expression of miR-451a and miR-20a-5p were observed between individuals with and without endometriosis.

MicroRNA-451a (miR-451a) is a conserved regulatory molecule intricately involved in cellular processes such as proliferation, apoptosis, and differentiation. Its involvement in various pathological conditions, including cancer, cardiovascular disease, and inflammatory disorders, is well documented. It also functions as a tumor suppressor, notably in neuroblastoma, osteosarcoma, and various cancers, including endometrial cancer, where its decreased expression is linked to an unfavorable prognosis (Cohn *et al.* 2010, Li *et al.* 2011*b*, Wang *et al.* 2011, Xu *et al.* 2014, Liu *et al.* 2016). miR-451a frequently acts as a tumor suppressor or anti-inflammatory regulator by targeting key signaling nodes such as IL-6R/JAK2/STAT3, MIF/PI3K/AKT, and transcription factors (ATF2, YWHAZ) (Shen *et al.* 2018, Wei *et al.* 2019, Zhong *et al.* 2020, Fu *et al.* 2021). These pathways are also implicated in endometriosis, where inflammation, angiogenesis, and lesion invasion are central pathogenic processes.

In the context of endometriosis, studies have indicated altered expression of miR-451a in ectopic lesions and peripheral blood, suggesting its potential role in its pathogenesis (Table S6) (Cosar *et al.* 2016, Nothnick *et al.* 2017, Moustafa *et al.* 2020). In endometriosis, the role of miR-451a remains controversial, with some studies reporting elevated expression in ectopic lesions or serum, while others describe reduced or unchanged levels (Li *et al.* 2011*a*, 2019, Graham *et al.* 2015, Joshi *et al.* 2015). In our study, using miR-16-5p as a stable reference (Fig. S1), we observed reduced circulating miR-451a levels in endometriosis patients, aligning more closely with tissue-based studies and supporting its possible role in the local disease microenvironment (Jia *et al.* 2013, Ravaggi *et al.* 2024).

miR-20a-5p, a member of the miR-17-92 cluster, regulates multiple cellular pathways, including inflammation, immunity, and tumorigenesis (Huang *et al.* 2022, Gao *et al.* 2024, Li *et al.* 2024). It has been shown to suppress proinflammatory cytokines via modulation of NF-κB signaling and is implicated in immune regulation (Zhao & Gong, 2019). Moreover, its expression varies across cancers, with context-specific roles in either promoting or inhibiting tumor progression (Huang *et al.* 2022). In endometrial cancer, miR-20a-5p functions as a tumor suppressor by targeting JAK1 and RUNX3 (He *et al.* 2021), while in endometriosis, it is frequently downregulated in both plasma and tissue, suggesting a potential role in immune evasion and lesion maintenance (Jia *et al.* 2013). Elevated expression of miR-20a has been implicated in the pathogenesis of ovarian endometriosis, primarily through the suppression of Netrin-4 (NTN4), a gene involved in cellular adhesion and angiogenesis (Zhao *et al.* 2014).

In our investigation, a reduction in the expression of circulating miR-20a-5p was observed in individuals with endometriosis compared to controls, consistent with findings from prior studies (Jia *et al.* 2013, Wang *et al.* 2016, Papari *et al.* 2020). The aforementioned studies utilized microarray or next-generation sequencing (NGS) technologies to determine the expression level of miR-20a-5p in the blood plasma/serum of endometriosis patients. These advanced technologies provide significant advantages, as they can generate unbiased, precise, consistent, and quantitative analyses of small RNA profiles. This further substantiates the reliability of the findings in our study despite the constraints posed by the small sample size.

Nevertheless, investigations conducted on endometrium and endometriotic tissue samples have presented contradictory findings regarding the differential expression of miR-20a-5p. Some studies indicate its downregulation (Ohlsson Teague *et al.* 2009, Filigheddu *et al.* 2010), while a few report its upregulation (Lin *et al.* 2012, Zhao *et al.* 2014, Kumari *et al.* 2021), and others show a mixed or no significant difference (Ramón *et al.* 2011), depending on the technology used for expression analysis between endometrium and control samples. Consequently, the current ambiguity raises questions about whether these disparities stem from variations in the selection of reference genes for normalization, differences in research methodologies, sample heterogeneity, or a potentially weak association between circulating miRNAs and miRNAs in endometrial tissues in the context of endometriosis.

Measuring total microRNAs presents ongoing technical challenges, highlighting the need for continued advancements in this area. Although miR-451a and miR-20a-5p have potential as diagnostic and prognostic biomarkers for endometriosis, various hurdles need to be overcome. Standardizing sample collection, RNA isolation methods, and miRNA quantification techniques is imperative for developing reproducible and dependable assays. Large-scale validation studies involving diverse patient cohorts are necessary to ascertain the clinical relevance and utility of these biomarkers.

Our study has several notable strengths. We conducted a comprehensive analysis of circulating fluids, particularly serum and plasma, to identify highly reliable and replicable miRNA targets. These targets underwent rigorous screening and validation, including evaluation in a different ethnic group with a high incidence of endometriosis. Unlike many studies that included control populations with various pelvic pathologies, we deliberately selected healthy controls without malignancies, thereby providing a distinct perspective. This study has certain limitations that warrant consideration. The primary limitation of this study is the relatively small sample size, which restricts the generalizability of the findings and limits the ability to explore subtype-specific associations. Since only advanced-stage patients were considered based on the American Society for Reproductive Medicine (ASRM) staging, the study’s ability to distinguish patients based on severity may be limited. This limitation also impedes the assessment of whether specific features of endometriosis, such as severity or stage, drive particular shifts in miRNA expression. While we controlled for hormonal variability by collecting all samples during the mid-luteal phase (days 18–24 of the menstrual cycle), we acknowledge that other potential confounders, such as body mass index (BMI) and lifestyle factors (including diet, exercise, and smoking habits), were not recorded. These variables may influence circulating miRNA expression and represent a limitation of the current study. Future investigations with more comprehensive clinical and lifestyle profiling are warranted to validate and refine these findings.

The search for reliable biomarkers based on miRNA levels has attracted significant attention in recent years. Despite numerous discoveries, more research is needed to enhance the consistency of miRNAs as disease markers. Moreover, there are still many unknowns regarding the physiological role of extracellular miRNAs and the mechanisms of their release and uptake by cells. As experimental techniques advance and our understanding of extracellular miRNAs improves, their use as noninvasive biomarkers will become invaluable. To further translate our findings to clinical practice, we would like to validate the efficacy of the identified biomarkers through large-scale studies across diverse patient groups to ensure reliability and reproducibility. Standardizing RNA extraction methods, miRNA quantification techniques, and normalization procedures is essential to achieve consistency across different laboratories. Evaluations of their clinical utility in diagnosing endometriosis, assessing disease severity, and predicting treatment outcomes require rigorous clinical trials. Furthermore, healthcare providers should be educated on interpreting and applying miRNA biomarkers in clinical settings and engaging patients in understanding their potential benefits and limitations. These steps collectively aim to facilitate the effective implementation of identified biomarkers, thereby improving endometriosis diagnosis and patient management.

A graphical abstract outlining the study workflow – from the literature review to sample selection, miRNA quantification, and biomarker identification – is provided as a separate file to aid in conceptual clarity and clinical translation.

## Conclusion

In our study, we explored circulating microRNAs as potential biomarkers for endometriosis in the Indian population. We identified significant findings related to miR-451a and miR-20a-5p, suggesting their relevance in diagnosing endometriosis. Our study highlighted the promise of using circulating microRNAs as noninvasive diagnostic markers, although standardization and larger validation cohorts are crucial due to discrepancies in miR-20a-5p expression across studies. Despite limitations such as sample size and clinical data, our comprehensive analysis across diverse ethnic groups supports the reliability of these findings and underscores the potential of microRNAs to transform endometriosis diagnosis and management.

## Supplementary materials



## Declaration of interest

The authors declare that there is no conflict of interest that could be perceived as prejudicing the impartiality of the work reported.

## Funding

This work was supported by grant no. F.8-A-769/2020/RS from the All India Institute of Medical Sciences (AIIMS), New Delhi, and grant no. 5/10/FR/57/2020-RBMCH from the Indian Council of Medical Researchhttps://doi.org/10.13039/501100001411 (ICMR), Government of India, to NK.

## Author contribution statement

SC performed work design, data acquisition, analysis, and interpretation of the data. AH and MS contributed to the conception and design of the work, and substantially revised the manuscript. JBS framed the inclusion and exclusion criteria, provided participant samples and helped in designing of the work. DP has contributed to conception, analysis, and drafting of the manuscript. NK contributed to conception, acquisition, analysis, and interpretation of data, and drafted the work. All authors read and approved the final manuscript.

## Ethics approval and consent to participate

The study was performed at the All India Institute of Medical Sciences (AIIMS), New Delhi, a tertiary care institution, after obtaining ethical approval from the Institutional Ethics Committee on 28th April 2022 (reference number IECPG-331/27.04.2022). Informed consent, both in English and in Hindi, was obtained from the participants.

## Data availability

All data generated or analyzed during this study are included in this published article and its supplementary information files, including Supplementary Figs 1 and 2, and Supplementary Tables 1 through 6.
