# Supplementary material for: Circulating microRNAs and endometriosis: a comprehensive analysis and validation of identified biomarkers in an Indian population
Source: Reprod Fertil. 2025 Oct 16;6(4):e250019. doi: 10.1530/RAF-25-0019 (PMC12538115; doi:10.1530/RAF-25-0019)
Supplement: Supplementary file 1 [file supplementary_materials.pdf]

Table S1 – Selected studies on circulating miRNA in endometriosis.

Sr. No	Year	Sample	Country	Technique	References
1	2013	Serum	China	qRT-PCR	Wang et al., 2013
2	2013	Plasma	China	Microarray profiling	Jia et al., 2013
3	2013	Plasma	USA	qRT-PCR	Suryawanshi et al., 2013
4	2015	Serum	South Korea	qRT-PCR	Cho et al., 2015
5	2015	Plasma	Spain	qRT-PCR	Rekker et al., 2015
6	2016	Serum	USA	Microarray profiling	Cosar et al., 2016
7	2018	Plasma	Iran	qRT-PCR	Bashti et al., 2018
8	2017	Serum	Egypt	qRT-PCR	Maged et al., 2018
9	2017	Serum	USA	qRT-PCR	Nematian et al., 2018
10	2016	Serum	China	NGS	Wang et al., 2016
11	2018	Serum	Italy	qRT-PCR	Di Pietro et al., 2018
12	2018	Plasma	Germany	qRT-PCR	Pateisky et al., 2018
13	2018	Plasma	China	QRT PCR	Wang et al., 2018
14	2019	Plasma	Australia	Microarray profiling	Nisenblat et al., 2019
15	2019	Plasma	Belgium	NGS	Vanhie et al., 2019
16	2020	Serum	China	qRT-PCR	Zhang et al., 2020
17	2020	Serum	USA	qRT-PCR	Moustafa et al., 2020
18	2020	Plasma	Iran	NGS, and qRT-PCR	Papari et al., 2020
19	2020	Serum	Turkey	qRT-PCR	Misir et al., 2021
20	2013	Serum	China	Microarray profiling	Hsu et al., 2014
21	2016	Serum	USA	qRT-PCR	Nothnick et al., 2017
22	2021	Plasma	Iran	qRT-PCR	Zafari et al., 2021

Table S2: Compilation of identified miRNAs associated with endometriosis in published literature.

Identified miRNA	[1]	[2]	[3]	[4]	[5]	[6]	[7]	[8]	[9]	[10]	[11]	[12]	[13]	[14]	[15]	[16]	[17]	[18]	[19]
miR-224-5p										1(-)								1(#)	
miR-199b-3p																			
let-7b-5p															1(#)		1(-)	1(#)	
miR-3613-5p						1(-)											1(-)	1(#)	
miR-125b-5p															1(-)		1(+)	1(-)	
miR-150-5p						1(+)											1(+)	1(#)	
miR-199a-5p										1(-)					1(#)				
miR-451a						1(+)						1(+)							
miR-34a-5p																			1(-)
miR-103a-3p															1(#)			1(#)	1(+)
miR-200c																			1(+)
miR-92a-3p															1(-)			1(-)	
miR-21-5p															1(-)			1(-)	
miR-17-5p		1(-)										1(-)			1(#)			1(#)	
miR-20a-5p										1(-)					1(#)			1(#)	
let-7a-3p										1(-)				1(#)				1(#)	
miR-486-5P														1(#)				1(#)	
let-7d-3p												1(-)						1(#)	
miR-143-3p						1(+)						1(+)						1(#)	
miR-381-3p												1(+)						1(#)	
miR-122-5p										1(-)								1(#)	
miR-335-3p										1(-)								1(#)	
miR-199a-3p																		1(#)	
miR-340-5p																		1(#)	
miR-221-3p																		1(#)	
miR-133a-3p																		1(#)	
miR-148a-5p																		1(#)	
miR-5																		1(#)	
miR-27																		1(#)	
miR-342-3p						1(+)				1(-)								1(+)	
miR-134-5p																1(-)			
miR-3141																1(-)			
miR-4499																1(-)			
miR-6088																1(-)			
miR-6165																1(-)			
miR-6728-5p																1(-)			
miR-22-3p															1(#)	1(+)			
miR-197-5p																1(+)			
miR-320a																1(+)			
miR-320b																1(+)			
miR-3692-5p																1(+)			
miR-4476																1(+)			
miR-4530																1(+)			

miR-4532								1(+)
miR-4721								1(+)
miR-4758-5p								1(+)
miR-494-3p								1(+)
miR-6126								1(+)
miR-6734-5p								1(+)
miR-6776-5p								1(+)
miR-6780b-5p								1(+)
miR-6785-5p								1(+)
miR-6791-5p								1(+)
miR-939-5p								1(+)
miR-98-5p					1(-)		1(#)	
miR-33a-5p					1(-)		1(#)	
miR-15b-5p		1(-)			1(+)		1(#)	
miR-101-3p					1(+)		1(#)	
let-7f-5p				1(-)			1(#)	
miR-182-5p				1(-)			1(#)	
miR-24-3p				1(-)			1(#)	
let-7a-5p				1(-)			1(#)	
miR-18a-5p			1(+)				1(#)	
let-7c-5p							1(#)	
let-7d-5p							1(#)	
let-7e-5p							1(#)	
miR-106a-5p							1(#)	
miR-10a-5p							1(#)	
miR-107							1(#)	
miR-148a-3p							1(#)	
miR-15a-5p							1(#)	
miR-16-5p							1(#)	
miR-17-3p							1(#)	
miR-199b-5p							1(#)	
miR-19a-3p							1(#)	
miR-19b-3p							1(#)	
miR-210-3p							1(#)	
miR-23a-3p							1(#)	
miR-25-3p							1(#)	
miR-26b-5p							1(#)	
miR-28-5p							1(#)	
miR-29a-3p							1(#)	
miR-29b-3p							1(#)	
miR-30a-3p							1(#)	
miR-30a-5p							1(#)	
miR-95-3p							1(#)	
miR-574-3p				1(-)		1(-)		
miR-9-5p				1(-)		1(-)		
miR-139-3p	1(-)					1(-)		

miR-135b						1(-)
miR-141-5p						1(-)
miR-155-5p						1(-)
miR-923						1(-)
miR-590-5p					1(-)	1(#)
miR-1					1(-)	1(#)
miR-331-3p					1(+)	1(#)
miR-654-3p	1(-)			1(-)		1(#)
miR-369-3p				1(-)		1(#)
miR-337-3p				1(-)		1(#)
miR-885-5p				1(-)		1(#)
miR-502-3p				1(+)		1(#)
miR-16			1(+)			1(#)
miR-19a		1(-)				1(#)
miR-19b		1(-)				1(#)
miR-630		1(-)				1(#)
miR-23a		1(-)				1(#)
miR-10b*	1(-)					1(#)
miR-598	1(-)					1(#)
miR-29c-3p						1(#)
let-7f-2						1(#)
let-7g						1(#)
miR-128						1(#)
miR-138-1*						1(#)
miR-130b						1(#)
miR-142-3p						1(#)
miR-143						1(#)
miR-146b						1(#)
miR-148a						1(#)
miR-148b						1(#)
miR-181c						1(#)
miR-183*						1(#)
miR-188-3p						1(#)
miR-200b						1(#)
miR-224						1(#)
miR-23b*						1(#)
miR-24						1(#)
miR-29a*						1(#)
miR-301b						1(#)
miR-30c						1(#)
miR-320						1(#)
miR-338-3p						1(#)
miR-361-5p						1(#)
miR-362-3p						1(#)
miR-376a*						1(#)
miR-378a*						1(#)

miR-422a				1(#)
miR-425				1(#)
miR-452				1(#)
miR-495				1(#)
miR-500				1(#)
miR-505				1(#)
miR-550*				1(#)
miR-532-5p				1(#)
miR-586				1(#)
miR-624*				1(#)
miR-625*				1(#)
miR-638				1(#)
miR-639				1(#)
miR-642				1(#)
miR-643				1(#)
miR-661				1(#)
miR-662				1(#)
miR-7-1*				1(#)
mir-874				1(#)
miR-93*				1(#)
miR-99b*				1(#)
miR-145-3p	1(-)			1(+)
miR-17			1(-)	
miR-326	1(-)	1(-)		
miR-625-3p	1(-)	1(-)		
miR-181d	1(-)	1(-)		
miR-183-5p	1(-)	1(-)		
miR-196b-5p	1(-)	1(-)		
miR-215	1(-)	1(-)		
miR-324-5p	1(-)	1(-)		
miR-296-5p	1(+)	1(-)		
miR-192-5p		1(-)		
miR-361-3p		1(-)		
miR-433		1(-)		
miR-497-5p		1(-)		
miR-7-5p		1(-)		
miR-22-5p		1(-)		
miR-421		1(-)		
miR-365a-3p		1(-)		
miR-193a-5p		1(-)		
miR-431-5p	1(-)	1(+)		
miR-485-3p	1(-)	1(+)		
miR-99b-5p	1(-)	1(+)		
miR-127-3p	1(-)	1(+)		
miR-134	1(-)	1(+)		
miR-154-5p		1(+)		

miR-375					1(+)
miR-133a					1(+)
miR-629-5p					1(+)
miR-32-5p					1(+)
miR-144-3p					1(+)
miR-378a-3p					1(+)
miR-339-5p					1(+)
miR-934					1(+)
miR-370					1(+)
miR-181a-5p					1(+)
miR-330-3p					1(+)
miR-185-3p					1(+)
miR-140-5p					1(+)
miR-27a-3p				1(+)	
miR-124-3p				1(+)	
miR-141-3p		1(-)	1(-)		
let-7c	1(-)		1(-)		
miR-328			1(-)		
miR-335-5p			1(-)		
miR-339-3p			1(-)		
miR-340-3p			1(-)		
miR-3605-5p			1(-)		
miR-3613-3p			1(-)		
miR-3614-5p			1(-)		
miR-374a-3p			1(-)		
miR-381			1(-)		
miR-382-5p			1(-)		
miR-409-3p			1(-)		
miR-410			1(-)		
miR-411-5p			1(-)		
miR-4286			1(-)		
miR-429			1(-)		
miR-432-5p			1(-)		
miR-4446-3p			1(-)		
miR-454-3p			1(-)		
miR-4772-5p			1(-)		
miR-4775			1(-)		
miR-485-5p			1(-)		
miR-487b			1(-)		
miR-493-5p			1(-)		
miR-5010-5p			1(-)		
miR-501-3p			1(-)		
miR-5683			1(-)		
miR-584-5p			1(-)		
miR-589-3p			1(-)		
miR-654-5p			1(-)		

miR-664-3p						1(-)
miR-744-5p						1(-)
miR-766-3p						1(-)
miR-877-5p						1(-)
miR-889						1(-)
miR-942						1(-)
miR-98						1(-)
let-7i-3p						1(-)
miR-100-5p						1(-)
miR-122-3p						1(-)
miR-1233						1(-)
miR-127-5p						1(-)
miR-1301						1(-)
miR-1307-3p						1(-)
miR-1307-5p						1(-)
miR-130b-5p						1(-)
miR-139-5p						1(-)
miR-150-3p						1(-)
miR-151a-5p						1(-)
miR-152						1(-)
miR-194-5p						1(-)
miR-20b-5p						1(-)
miR-214-3p						1(-)
miR-221-5p						1(-)
miR-222-3p						1(-)
miR-26a-5p						1(-)
miR-27b-5p						1(-)
miR-29c-5p						1(-)
miR-30b-5p						1(-)
miR-30c-5p						1(-)
miR-3155a						1(-)
miR-31-5p						1(-)
miR-3163						1(-)
miR-3200-5p						1(-)
miR-323a-3p						1(-)
miR-542-3p	1(-)					1(+)
miR-185-5p						1(+)
miR-242-5p						1(+)
miR-3127-5p						1(+)
miR-424-3p						1(+)
miR-4645-3p						1(+)
miR-550a-3p						1(+)
miR-636						1(+)
let-7b	1(-)		1(-)		1(-)	
miR-125b					1(+)	
miR-122	1(+)	1(-)		1(+)		

miR-199a	1(+)							1(+)
miR-31							1(-)	
miR-145							1(+)	
miR-6755-3p						1(-)		
miR-500a-3p						1(+)		
miR-145-5p						1(+)		
miR-200a-3p					1(-)			
miR-200b-3p					1(-)			
let-7d				1(-)				
let-7e				1(-)				
let-7f				1(-)				
miR-135a				1(-)				
let-7a				1(#)				
miR-135b				1(#)				
miR-195			1(+)					
miR-191			1(+)					
miR-1974			1(+)					
miR-4284			1(+)					
miR-15b			1(+)					
miR-1978			1(+)					
miR-1979			1(+)					
miR-362-5p			1(+)					
miR-1973			1(+)					
miR-572	1(-)	1(-)						
miR-20a		1(-)						
miR-451		1(-)						
miR-30e		1(-)						
miR-27a		1(-)						
miR-92a		1(-)						
miR-26a		1(-)						
miR-320e		1(-)						
miR-320d		1(-)						
miR-762		1(-)						
miR-1274b		1(-)						
miR-29c		1(-)						
miR-223		1(-)						
miR-22		1(-)						
miR-1268		1(-)						
miR-H10		1(-)						
miR-21		1(-)						
miR-3679-5p		1(-)						
miR-30d		1(-)						
mir-3665		1(-)						
miR-141*	1(-)							
miR-489	1(-)							
miR-1243	1(-)							

miR-145*	1(-)
miR-9*	1(-)
miR-128a	1(-)
miR-548b-5p	1(-)
miR-758	1(-)
miR-202	1(-)
miR-20a*	1(-)
miR-18a*	1(-)
miR-19b-1*	1(-)
miR-185	1(-)
miR-511	1(-)
miR-335*	1(-)
miR-515-3p	1(-)
miR-548c-5p	1(-)
miR-27a*	1(-)
miR-645	1(-)
miR-324-3p	1(-)
miR-204	1(-)
miR-886-5p	1(-)
miR-384	1(-)
miR-296	1(-)
miR-323-3p	1(-)
miR-206	1(-)
miR-520c-3p	1(-)
miR-1291	1(-)
miR-1825	1(-)
miR-337-5p	1(+)
miR-378	1(+)
miR-193a-3p	1(+)
miR-504	1(+)
miR-708	1(+)
miR-130a	1(+)
miR-330-5p	1(+)
miR-218-1*	1(+)
miR-1180	1(+)
miR-520d-3p	1(+)
miR-561	1(+)
miR-203	1(+)
miR-365	1(+)
miR-1275	1(+)
miR-377*	1(+)
miR-302a	1(+)
miR-647	1(+)
miR-455	1(+)
miR-367	1(+)
miR-651	1(+)

miR-122*	1(+)

"+" indicates upregulated miRNAs, "–" indicates downregulated miRNAs, and "#" denotes no significant change. References correspond to the studies listed in Table S1.

Table S3. Compilation of validated miRNAs associated with endometriosis in published literature

Validated miRNA	[1]	[2]	[3]	[4]	[6]	[10]	[12]	[14]	[15]	[16]	[17]	[18]	Upregulated (Total)	Downreg (Total)
miR-21-5p									1(#)			1(-)		1(-)
miR-125b-5p					1(+)				1(#)		1(+)		2(+)	
miR-28-5p									1(#)					
miR-29a-3p									1(#)					
let-7d-5p									1(#)					
miR-30a-5p									1(#)					
miR-20a-5p						1(-)						1(-)		2(-)
miR-17-5p		1(-)										1(-)		2(-)
miR-199a-3p												1(-)		2(-)
miR-3613-5p					1(-)						1(-)			2(-)
let-7b				1(-)							1(-)			2(-)
miR-143-3p					1(+)							1(-)	1(+)	1(-)
miR-340-5p												1(-)		1(-)
let-7b-5p												1(-)		1(-)
miR-103a-3p												1(-)		1(-)
miR-134-5p										1(-)				1(-)
miR-155								1(-)						1(-)
miR-574-3p								1(-)						1(-)
miR-139-3p								1(-)						1(-)
miR-33a-5p							1(-)							1(-)
miR-196b-5p							1(-)							1(-)
miR-30c-5p						1(-)								1(-)
miR-127-3p						1(-)								1(-)
miR-99b-5p						1(-)								1(-)
miR-15b-5p						1(-)								1(-)
miR-6755-3p					1(-)									1(-)
miR-135a				1(-)										1(-)
miR-20a		1(-)												1(-)

miR-22		1(-)								1(-)
miR-145*	1(-)									1(-)
miR-141*	1(-)									1(-)
miR-542-3p	1(-)									1(-)
miR-9*	1(-)									1(-)
miR-22-3p							1(+)		1(+)	
miR-320a							1(+)		1(+)	
miR-197-5p							1(+)		1(+)	
miR-494-3p							1(+)		1(+)	
miR-939-5p							1(+)		1(+)	
miR-154-5p						1(+)			1(+)	
miR-378a-3p						1(+)			1(+)	
miR-424-3p					1(+)				1(+)	
miR-185-5p					1(+)				1(+)	
miR-500a-3p				1(+)					1(+)	
miR-18a-5p				1(+)					1(+)	
miR-145-5p				1(+)					1(+)	
miR-16			1(+)						1(+)	
miR-195			1(+)						1(+)	
miR-191			1(+)						1(+)	
miR-199a	1(+)								1(+)	
miR-122	1(+)								1(+)	
miR-451a				1(+)				1(+)	2(+)	
miR-150-5p				1(+)				1(+)	2(+)	
miR-342-3p				1(+)				1(+)	2(+)	

“+” indicates upregulated miRNAs, “–” indicates downregulated miRNAs, and “#” denotes no significant change. References correspond to the studies listed in Table S1.

Table S4. Final Identified and Validated miRNAs based on replicated at least in two studies

Identified (Replicated)		Validated (Replicated)	
**Up**	**Down**	**Up**	**Down**
miR-451a	let-7b	miR-125b-5p	miR-17-5p
miR-122	miR-125b-5p	miR-451a	let-7b
miR-342-3p	miR-17-5p	miR-150-5p	miR-3613-5p
miR-143-3p	miR-3613-5p	miR-342-3p	miR-20a-5p
miR-150-5p	miR-654-3p		miR-199a-3p
miR-199a	let-7d-3p		
	miR-224-5p		
	miR-92a-3p		
	miR-572		
	let-7c		
	miR-141-3p		
	miR-326		
	miR-574-3p		
	miR-625-3p		
	miR-181d		
	miR-183-5p		
	miR-196b-5p		
	miR-215		
	miR-324-5p		
	miR-9-5p		
	miR-486-5P		
	miR-21-5p		

“+” indicates upregulated miRNAs, “–” indicates downregulated miRNAs, and “#” denotes no significant change. References correspond to the studies listed in Table S1.

Table S5: Study subjects characteristics.

Characteristic	Endometriosis	Controls	P-value
Sample Numbers	12	11	
Mean Age ± SD (years)	31.9 ± 6.0 (24-39)	28 ± 5.8 (22-37)	0.1663
Surgical inspection of pelvis/abdomen, n (%)	12 (100%)	Nil	
**Clinical Presentations**			
Dysmenorrhea	10 (83%)	Nil	
Menorrhagia	6 (50%)	Nil	
Dysmenorrhea+Menorrhagia	5 (42%)	Nil	
Pain + infertility	9 (75%)	Nil	
Painful micturition	2(16%)	Nil	
Irregular Menstral Cycle	2 (16%)	Nil	
Fertility#	3 (25%)	11 (100%)*	
**Diagnosis method**			
Laparoscopy	12 (100%)	Nil	
MRI	5 (42%)	Nil	
CA-125 (U/ml)	51.9 ± 9.5	16.0 ± 8.0	<0.0001
Estradiol (E2), (pg/ml)	139.7 ± 36.9	144.1± 34.1	0.7689
Progesterone (ng/ml)	10.3 ± 2.9	10.6 ± 3.2	0.8557

* In all selected controls, the most recent childbirth occurred less than two years prior, with each participant having delivered at least one baby.

# Three patients had at least one child following spontaneous conception.

Table S6: Comprehensive overview of miR-451a.

Year	Sample	Country	Sample Number	Reference gene	Expression level	AUC
2011	Tissue	China	Elevated P = 12, Normal P = 7	ACTB	Down (Normal P)	NA
2015	Tissue	USA	E = 41, C = 30	U6	Up	NA
2015	Tissue	USA	E = 19 C = 7	RPS17	Down	NA
2016	Serum	USA	E = 24, C = 24	U6	Up	0.835
2017	Serum	USA	E = 41, C = 40	U6	Up	0.8599
2019	Follicular Fluid	China	E = 30 C = 30	U6	Down	NA
2020	Serum	USA	E = 41, C = 59	U6	Up	0.84
2024	Plasma	India	E =12, C = 11	has-miR-16	Down	0.8939

P indicates progesterone, E-Endometriosis, C-Control

Figure S1: Ct Values of miR-16-5p in Plasma Samples from Endometriosis Patients and Controls. Raw cycle threshold (Ct) values of miR-16-5p used to assess its suitability as an endogenous reference gene for normalization in plasma miRNA analysis.

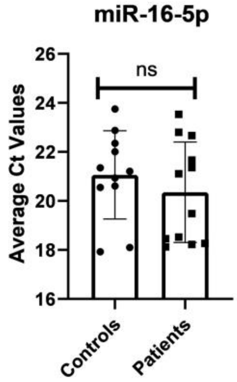

Figure S2: ROC analysis and diagnostic performance of the logistic regression model using miR-20a-5p and miR-451a for endometriosis classification. A) ROC curve illustrating the classification performance of the logistic regression model for endometriosis diagnosis. The curve was generated using predicted probabilities obtained from the logistic regression analysis. B) Summary of diagnostic performance metrics for the logistic regression model. The table includes AUC, cut-off value, sensitivity, specificity, and statistical significance. AUC was derived from ROC analysis, and performance measures were calculated at the specified probability threshold.

A

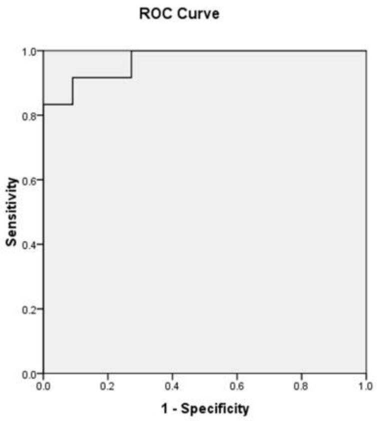

B

Model	AUC (95% CI)	Cut-off Value	Sensitivity	Specificity	p-value
miR-20a-5p + miR-451a (LogReg)	0.97	0.5 (default)	91.70%	81.80%	0.000
